# Seven key hub genes identified by gene co-expression network in cutaneous squamous cell carcinoma

**DOI:** 10.1186/s12885-021-08604-y

**Published:** 2021-07-23

**Authors:** Huizhen Chen, Jiankang Yang, Wenjuan Wu

**Affiliations:** 1 School of Basic Medical Sciences, Dali University, Dali, Yunnan 671000 People’s Republic of China; 2 Institute of Translational Medicine for Metabolic Diseases, Dali University, Dali, Yunnan 671000 People’s Republic of China; 3 Department of Dermatology, First Affiliated Hospital of Kunming Medical University, Kunming, Yunnan 650000 People’s Republic of China

**Keywords:** Cutaneous squamous cell carcinoma, Hub gene, Weighted gene co-expression network analysis, Survival

## Abstract

**Background:**

Cutaneous squamous cell carcinoma (cSCC) often follows actinic keratosis (AK) and is the second most common skin cancer worldwide. To reduce metastasis risk, it is important to diagnose and treat cSCC early. This study aimed to identify hub genes associated with cSCC and AK.

**Methods:**

This study used three datasets GSE45216, GSE98774, and GSE108008. We combined samples from the GSE45216 and GSE98774 datasets into the new dataset GSE45216–98774. We applied a weighted gene co-expression network analysis (WGCNA) to investigate key modules and hub genes associated with cSCC and AK. We considered the hub genes found in both the GSE45216–98774 and GSE108008 datasets as validated hub genes. We tested whether the expression of hub genes could predict patient survival outcomes in other cancers using TCGA pan-cancer data.

**Results:**

We identified modules most relevant to cSCC and AK. Additionally, we identified and validated seven hub genes of cSCC: *GATM*, *ARHGEF26*, *PTHLH*, *MMP1*, *POU2F3*, *MMP10* and *GATA3*. We did not find validated hub genes for AK. Each hub gene was significantly associated with the survival of various cancer types. Only *GATA3* was significantly associated with melanoma survival.

**Conclusions:**

We applied WGCNA to find seven hub genes that play important roles in cSCC tumorigenesis. These results provide new insights that help explain the pathogenesis of cSCC. These hub genes may become biomarkers or therapeutic targets for accurate diagnosis and treatment of cSCC in the future.

**Supplementary Information:**

The online version contains supplementary material available at 10.1186/s12885-021-08604-y.

## Background

Cutaneous squamous cell carcinoma (cSCC) is the second most common skin cancer after basal cell carcinoma, and in recent years has experienced increased incidence. cSCC is prone to metastasis. The transition to invasive cSCC has been reported to occur in 10% of diagnosed cases [1]. Frequent moderate chronic ultraviolet irradiation exposure can cause cSCC. cSCC development usually follows actinic keratosis (AK), a small, rough raised area on the skin which is, in most cases, the precursor lesion of cSCC. To reduce metastasis risk, it is important to diagnose and treat cSCC early; however, there are no clinically useful biomarkers for cSCC yet.

The pathogenesis of cSCC involves multiple genetic alterations that may dysregulate cell function. Recent genome-wide association studies from patients identified several loci associated with cSCC, including pigmentation-related loci [2, 3]. Many studies have attempted to understand the mechanism of cSCC by comparing cSCC and normal samples through differential gene expression analysis. These studies found differentially expressed genes (DEGs) involved mainly in cell division, cell cycle, apoptosis, inflammation, and epidermal differentiation [4–6].

Weighted gene co-expression network analysis (WGCNA) consists of constructing weighted correlation networks to identify high correlations between key modules and clinical traits. Also, WGCNA can measure relationships between modules and genes, even ranking genes within modules. It is, therefore, a useful tool to perform association analyses of gene sets with diseases and identify candidate hub genes [7, 8]. Cancer research has extensively used WGCNA [9]. A study on pancreatic ductal adenocarcinoma using WGCNA identified 5 modules and found 10 hub genes that may indicate a poor prognosis [10].

In this study, we applied this method to identify key modules and hub genes associated with cSCC and AK. We also tested whether the expression of hub genes could predict survival outcomes in other cancers using TCGA pan-cancer data.

## Methods

### Data collection

**Fig. 1:**
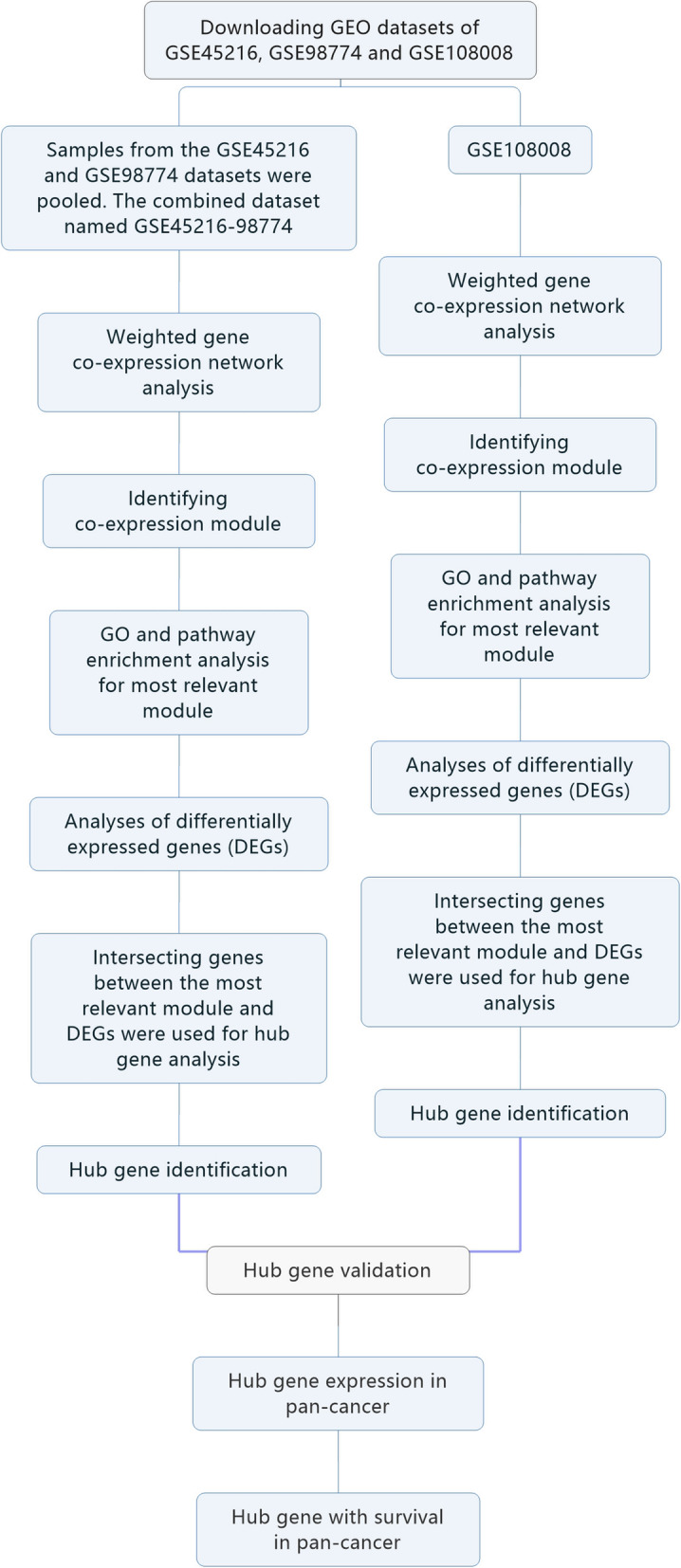
Flow diagram of the whole analysis procedure

The flow chart in Fig. 1 illustrates the procedures used in our study. We obtained the normalized, scaled, and pre-processed array data for cSCC from the GEO database (https://www.ncbi.nlm.nih.gov/geo/). The GSE45216 dataset has 30 cSCC samples and 10 AK samples. The GSE98774 dataset has 18 AK samples and 36 normal samples. We pooled samples from the GSE45216 and GSE98774 datasets. We applied Combat over GSE45216 and GSE98774 to correct for batch effects [11]. The combined dataset, named GSE45216–98774, contained 30 cSCC samples, 28 AK samples, and 36 normal samples. The GSE108008 dataset consists of 10 cSCC samples, 10 AK samples, and 10 normal samples. These two datasets, GSE45216–98774 and GSE108008, would be used for the subsequent analysis, respectively. The results of the analysis were mutually verified.

### Weighted gene co-expression network analysis

We constructed gene co-expression networks using the WGCNA package of R [12]. First, we removed unexpressed genes based on the expression profile and calculated the variance for each gene. We selected the genes with standard deviations in the top 50% for further analysis. Some samples were distant, and we excluded outliers based on cluster distance. To construct a weighted gene network, we set the soft threshold power β to five for GSE45216–98774 and six for GSE108008, which were the lowest power based on a scale-free topology [13]. After constructing a scale-free network, we transformed the expression matrix into an adjacency matrix and a topological matrix. On the basis of the topological overlap measure, we used the average-linkage hierarchical clustering method to cluster genes and set the minimum number of genes per module to 30. We set the threshold for similar module combinations to 0.25. After identifying gene modules using dynamic shear, we calculated module eigengenes (MEs, the first principal component of one module), and then clustered modules and merged closer modules into new modules based on height = 0.25. To identify associations between modules and clinical characteristics, we plotted a heat map of modules-characteristics relationship. We selected genes in the most significant module for subsequent analysis.

### Functional enrichment analysis of network module genes

To analyze the genes in modules at the functional level, we performed Gene Ontology (GO) [14] and Kyoto Encyclopedia Gene and Genomes (KEGG pathway) [15] enrichment analyses using the cluster-Profiler package [16]. We identified overrepresented GO terms and KEGG pathways. We chose 0.05 as the threshold for the false discovery rate (FDR) adjusted q-value.

### Analyses of DEGs

We identified the DEGs using the limma package [17]. We fitted a linear model to each gene and assessed the expression differences using empirical Bayes moderated t-statistics. We estimated the FDR adjusted q-value. Statistical significance for differential expression was set to q-value < 0.05, coupled with a |log2 fold change (log2FC)| > 1.

### Identification of hub gene and validation

We used the intersecting genes between the most relevant module and DEGs for hub gene analysis. Hub genes are a class of highly connected genes within a module and are significantly associated with biological function [18]. In this study, we defined genes with high module membership (MM) (|cor.weighted| > 0.8) as hub genes. We considered hub genes present in both GSE45216–98774 and GSE108008 as validated hub genes.

### Hub gene expression in pan-cancer

TCGA pan-cancer data, including RNA-Seq (RNAseq-FPKM) and clinical data, were downloaded from xena browser (https://xenabrowser.net/datapages/) [19]. The TCGA pan-cancer data include 33 cancer types, and they are adrenocortical carcinoma (ACC), bladder Urothelial Carcinoma (BLCA), breast invasive carcinoma (BRCA), cervical squamous cell carcinoma and endocervical adenocarcinoma (CESC), cholangio carcinoma (CHOL), colon adenocarcinoma (COAD), lymphoid neoplasm diffuse large B-cell lymphoma (DLBC), esophageal carcinoma (ESCA), glioblastoma multiforme (GBM), head and neck squamous cell carcinoma (HNSC), kidney chromophobe (KICH), kidney renal clear cell carcinoma (KIRC), kidney renal papillary cell carcinoma (KIRP), acute myeloid leukemia (LAML), brain lower grade glioma (LGG), liver hepatocellular carcinoma (LIHC), lung adenocarcinoma (LUAD), lung squamous cell carcinoma (LUSC), mesothelioma (MESO), ovarian serous cystadenocarcinoma (OV), pancreatic adenocarcinoma (PAAD), pheochromocytoma and paraganglioma (PCPG), prostate adenocarcinoma (PRAD), rectum adenocarcinoma (READ), sarcoma (SARC), skin cutaneous melanoma (SKCM), stomach adenocarcinoma (STAD), testicular germ cell Tumors (TGCT), thyroid carcinoma (THCA), thymoma (THYM), uterine corpus endometrial carcinoma (UCEC), uterine carcinosarcoma (UCS), and uveal melanoma (UVM). Comparison of gene expression between the normal samples and tumors was performed in 21 cancer types which had more than three associated adjacent normal samples using Wilcox statistical test. Statistical significance for differential expression was set to *P* < 0.05, coupled with a |(log2FC)| > 1.

### Association of hub gene with patient overall survival in pan-cancer

To investigate the association between hub genes and patient overall survival, all patient tumor samples were used in a survival analysis. Patients were stratified into a high-level group or a low-level group according to the median expression level, and the Kaplan–Meier method was used to analyze survival. *P* < 0.05 indicates significant differences.

## Results

### A weighted gene co-expression network

**Fig. 2:**
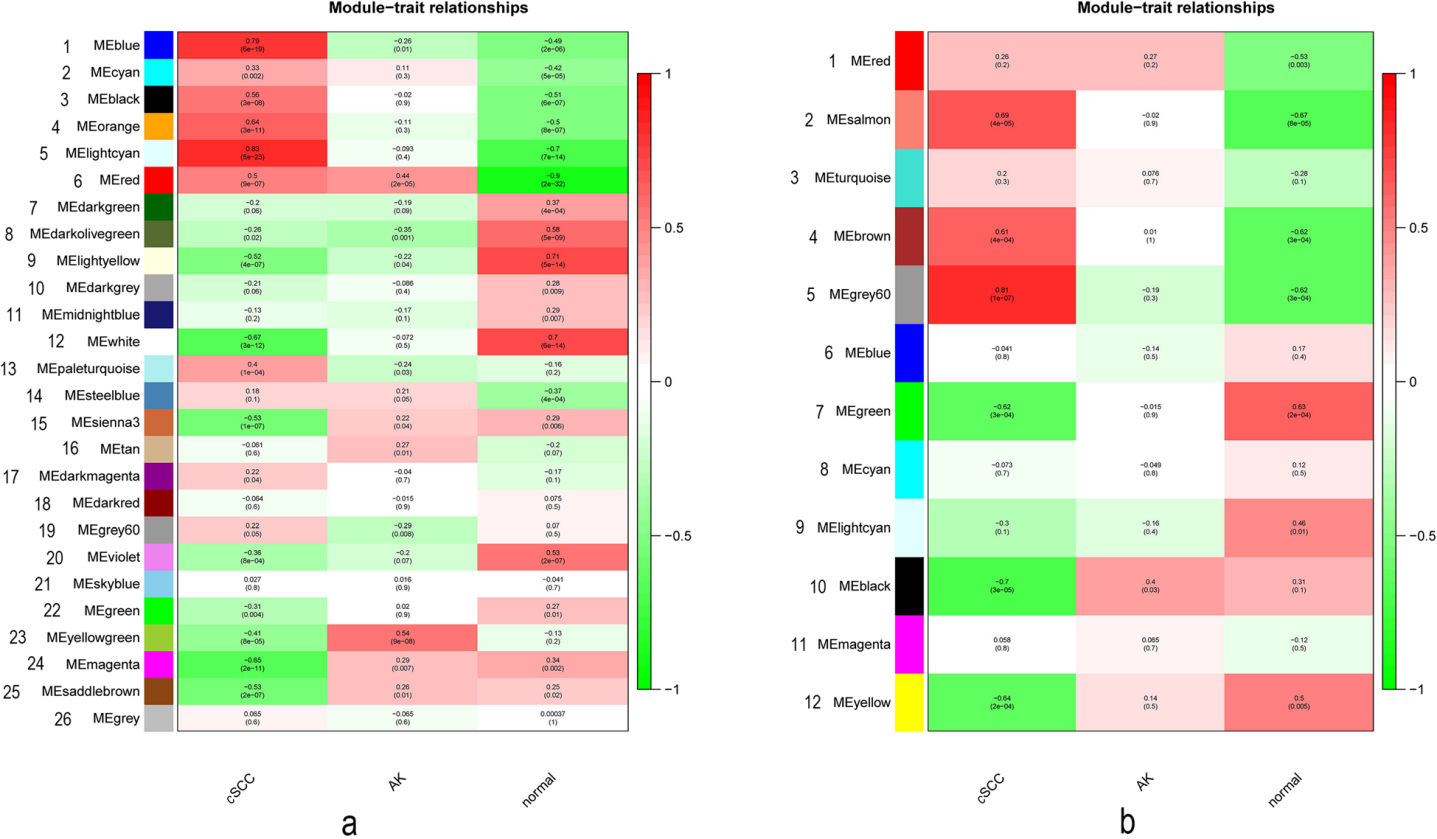
Identification of modules associated with cSCC, AK and normal samples. **a** Heatmap of the correlations between relevant modules and clinical characteristics for the GSE45216–98774 dataset. **b** Heatmap of the correlations between relevant modules and clinical characteristics for the GSE108008 dataset

We identified a total of 26 modules in the GSE45216–98774 dataset and highlighted these separately with different colors. We calculated correlations between modules and clinical characteristics, as shown in Fig. 2a. According to the correlation between MEs and characteristics, module 5 was the most relevant for cSCC. This module contained 1742 genes (Supplementary Table 1). Module 23 was the most relevant for AK and included 31 genes (Supplementary Table 2). Module 9 was the most relevant for normal samples and included 352 genes (Supplementary Table 3).

We identified a total of 12 modules in the GSE108008 dataset and highlighted them separately with different colors. We calculated the correlations between modules and clinical characteristics, as shown in Fig. 2b. Module 5 was the most relevant for cSCC and had 249 genes (Supplementary Table 4). Module 10 was the most correlated module for AK and contained 324 genes (Supplementary Table 5). Module 7 was the most correlated module for normal samples and included 525 genes (Supplementary Table 6). Next, these modules would be further analyzed.

### Gene ontology and pathway enrichment analyses

**Fig. 3:**
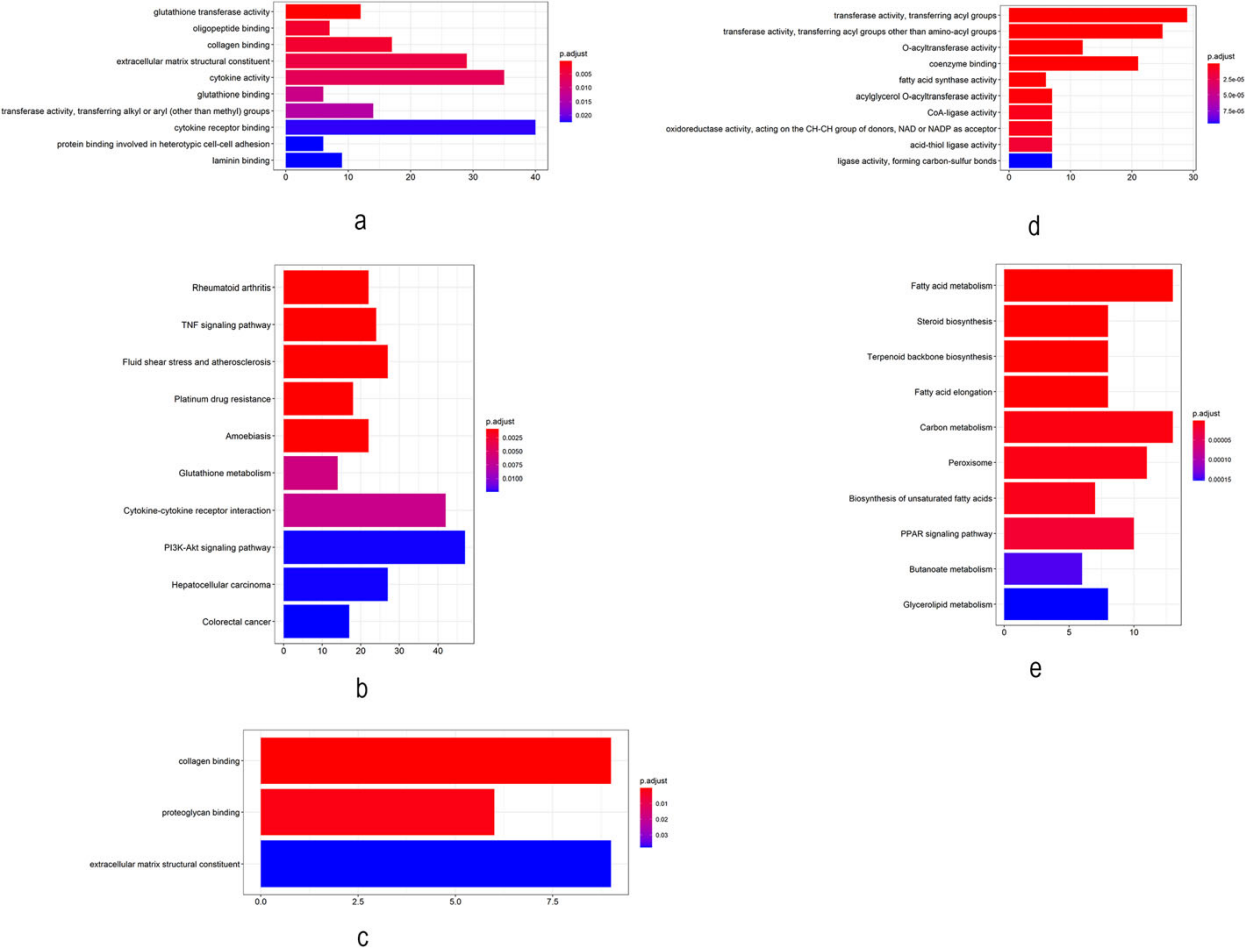
Functional enrichment for modules most associated with cSCC and AK. **a** Most overrepresented GO terms in module 5 (relevant for cSCC) for the GSE45216–98774 dataset. **b** KEGG functional enrichment of genes in module 5 (relevant for cSCC) for the GSE45216–98774 dataset. **c** GO functional enrichment of genes in module 5 (relevant for cSCC) for the GSE108008 dataset. **d** Most overrepresented GO terms in module 10 (relevant for AK) for the GSE108008 dataset. **e** KEGG functional enrichment of genes in module 10 (relevant for AK) for the GSE108008 dataset

GO and KEGG pathway enrichment analyses were performed for genes in the module 5 (relevant for cSCC) of the GSE45216–98774 dataset. The most overrepresented GO terms are listed in Fig. 3a and Supplementary Table 7. They were associated with glutathione, collagen, extracellular matrix and cytokine, among others. According to the KEGG database, the genes in module 5 were mainly enriched in the TNF signaling pathway, glutathione metabolism, cytokine-cytokine receptor interaction, hepatocellular carcinoma, colorectal cancer and focal adhesion, among others (Fig. 3b and Supplementary Table 8). GO and KEGG pathway enrichment analyses were performed for genes in module 23 (relevant for AK). No overrepresented GO term with an adjusted q < 0.05 was found. According to the KEGG database, genes in the module 23 were mainly enriched in one pathway of mineral absorption (Supplementary Table 9). GO and KEGG pathway enrichment analyses were also performed for genes in module 9 (relevant for normal samples). Overrepresented GO terms were associated with scavenger receptor activity, among others (Supplementary Table 10). According to the KEGG database, genes in module 9 were mainly enriched in Wnt signaling pathway and complement and coagulation cascades (Supplementary Table 11).

GO and KEGG pathway enrichment analyses were performed for genes in module 5 (relevant for cSCC) of the GSE108008 dataset. Figure 3c and Supplementary Table 12 list the top overrepresented GO terms. Notably, they were associated with collagen and extracellular matrix, among others. According to the KEGG database, genes in module 5 were mainly enriched in one pathway of focal adhesion (Supplementary Table 13). We performed GO and KEGG pathway enrichment analyses for genes in module 10 (related to AK). The most overrepresented GO terms were associated with key enzymes in controlling the synthesis of fatty acid and triglycerides, among others (Fig. 3d and Supplementary Table 14). According to the KEGG database, genes in the module 10 were mainly enriched for fatty acid metabolism, steroid biosynthesis and terpenoid backbone biosynthesis, among others (Fig. 3e and Supplementary Table 15). We also performed GO and KEGG pathway enrichment analyses for genes in module 7 (related to normal samples). The most overrepresented GO terms were associated with sulfur compound binding, Wnt-protein binding, extracellular matrix structural constituent and fibronectin binding, among others (Supplementary Table 16). According to the KEGG database, genes in module 7 were mainly enriched for Wnt signaling pathway (Supplementary Table 17).

According to the results of KEGG pathway enrichment for cSCC, focal adhesion was enriched in both datasets. Focal adhesions are composed of adhesins outside the cell membrane, integrins on the cell membrane and cytoskeletal proteins in the cell. The functions of focal adhesion are mechanical structure function and signal transmission function. Abnormalities in this pathway may be one of the causes of cSCC pathogenesis. Wnt signaling pathway for normal tissue was enriched in both datasets. Wnt signaling is a key pathway in controlling skin development and homeostasis, which indicates the importance of this pathway for the maintenance of normal skin function. As for AK, the two datasets were not enriched in common pathways associated with AK.

### Detection of DEGs

**Fig. 4:**
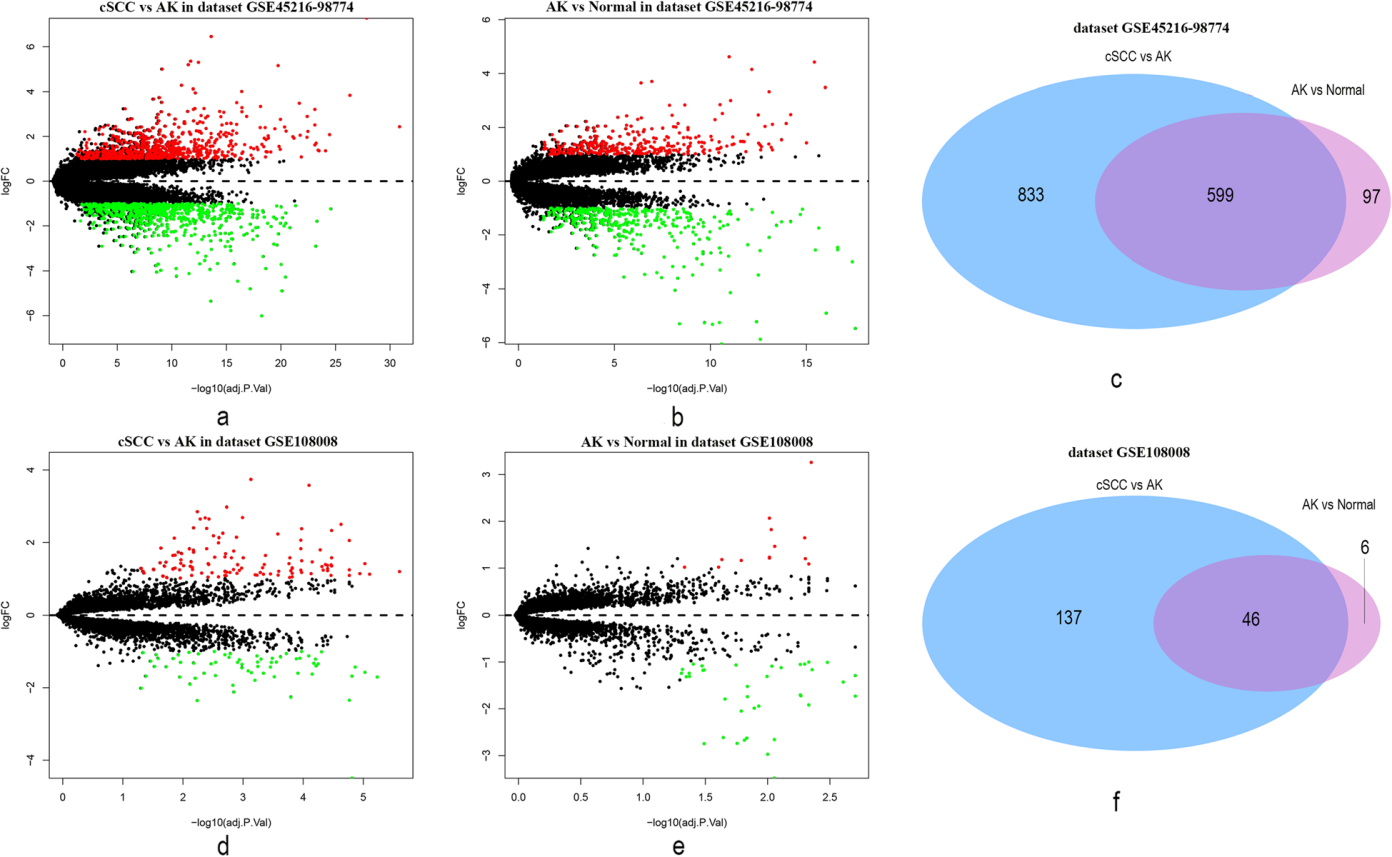
Volcano plots and Venn diagrams reflecting significant differentially expressed genes (DEGs). **a** Comparison between cSCC and AK samples from the GSE45216–98774 dataset uncovered 1432 DEGs. **b** Comparison between AK and normal samples from the GSE45216–98774 dataset uncovered 696 DEGs. **c** Venn diagram showing the common genes in comparisons between the GSE45216–98774 dataset samples (cSCC vs AK samples and AK vs normal samples). **d** Comparisons between cSCC and AK samples from the GSE108008 dataset uncovered 183 DEGs. **e** Comparisons between AK and normal samples from the GSE108008 uncovered 52 DEGs. **f** Venn diagram showing the common genes in comparisons between the GSE108008 dataset samples (cSCC vs AK samples and AK vs normal samples)

By comparing the cSCC and AK samples in the GSE45216–98774 dataset, we identified a total of 1432 DEGs (Fig. 4a and Supplementary Table 18), including 722 down-regulated genes and 710 up-regulated genes. By comparing AK and normal samples, we found 696 DEGs (Fig. 4b and Supplementary Table 19). Among them, 377 genes were down-regulated and 319 genes were up-regulated. We identified 599 relevant DEGs present in all the comparisons, indicating that they may play continuous roles in AK and cSCC development (Fig. 4c).

By comparing cSCC and AK samples in the GSE108008 dataset, we identified a total of 183 DEGs (Fig. 4d and Supplementary Table 20), including 65 down-regulated genes and 118 up-regulated genes. By comparing AK and normal samples, we found 52 DEGs (Fig. 4e and Supplementary Table 21). Of these, 39 were down-regulated and 13 were up-regulated. We identified 46 relevant DEGs present in all the comparisons, indicating that they may play continuous roles in AK and cSCC development (Fig. 4f). Among them, 18 genes were present in both the GSE45216–98774 and GSE108008 datasets, including *ACER1*, *ACSBG1*, *ACSL1*, *APOD*, *CST6*, *CYB5A*, *DGAT2*, *ELOVL4*, *FAXDC2*, *IL24*, *MET*, *MMP1*, *MMP10*, *MMP12*, *PLEK2*, *PSAPL1*, *PTHLH* and *STC1*. Thus, these genes have a relatively high priority for future research.

### Hub gene identification and validation

In the GSE45216–98774 dataset, 418 DEGs (from the cSCC/AK samples comparison) were also present in module 5 (relevant for cSCC). Ten DEGs (from the AK/normal samples comparison) were also present in module 23 (relevant for AK). One gene from module 23 and 51 genes from module 5 fulfilled the screening criteria of hub genes in the co-expression network.

In the GSE108008 dataset, 21 DEGs (from the cSCC/AK samples comparison) were also present in module 5 (relevant for cSCC). Thirty-two DEGs (from the AK/normal samples comparison) were also present in module 10 (relevant for AK). Sixteen genes from module 5 and 30 genes from module 10 fulfilled the criteria for hub genes in the co-expression network.

**Fig. 5:**
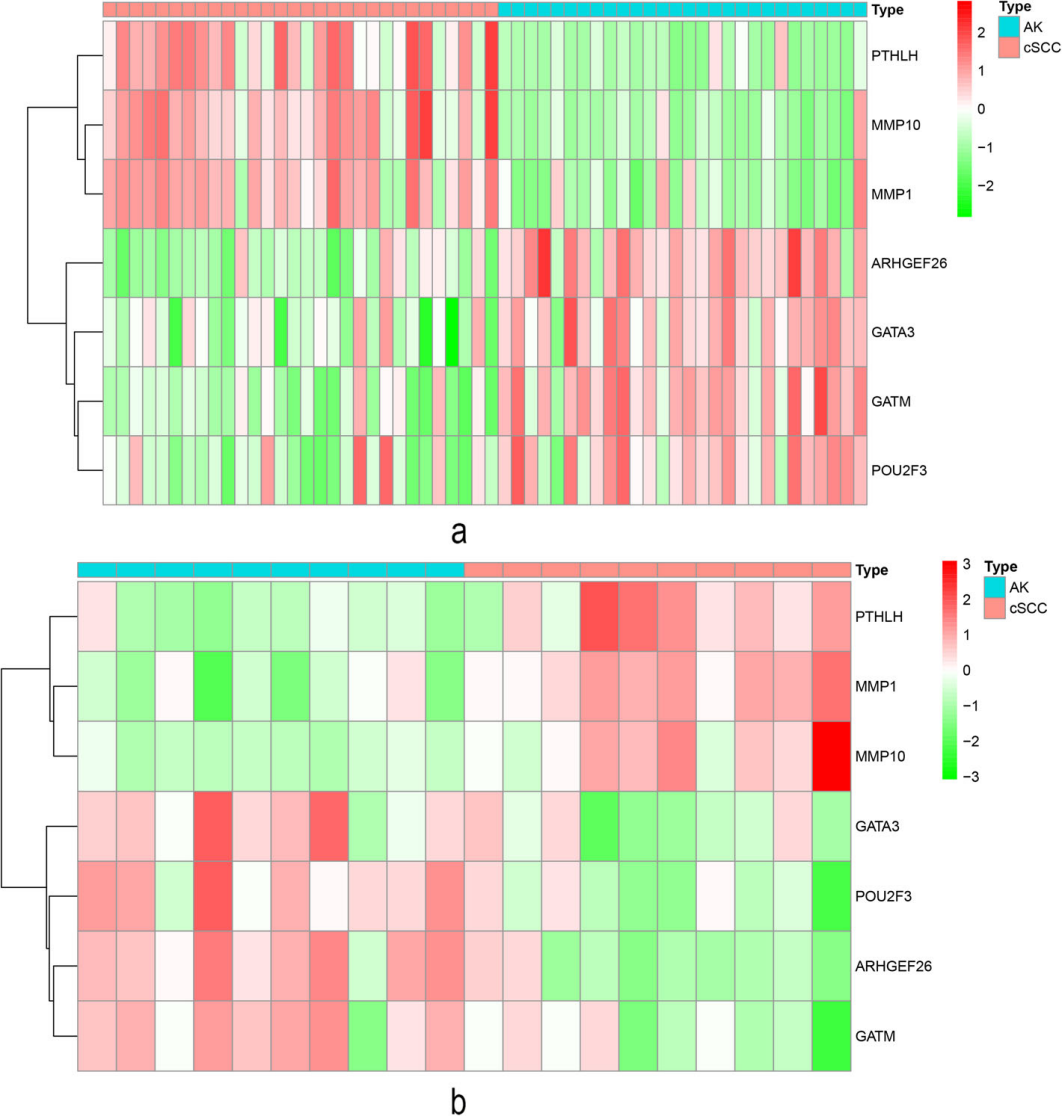
Differentially expressed heatmaps of the seven validated hub genes between cSCC samples and AK samples. **a** Heatmap for the GSE45216–98774 dataset. **b** Heatmap for the GSE108008 dataset

We considered the hub genes present in both the GSE45216–98774 and GSE108008 datasets as validated hub genes. Finally, there were seven hub genes for cSCC, including *GATM*, *ARHGEF26*, *PTHLH*, *MMP1*, *POU2F3*, *MMP10* and *GATA3*. We visualized the expression of these seven hub genes between cSCC and AK samples by plotting heatmaps (Fig. 5a and b). However, no hub gene was validated for AK.

### Hub gene expression in pan-cancer

**Fig. 6:**
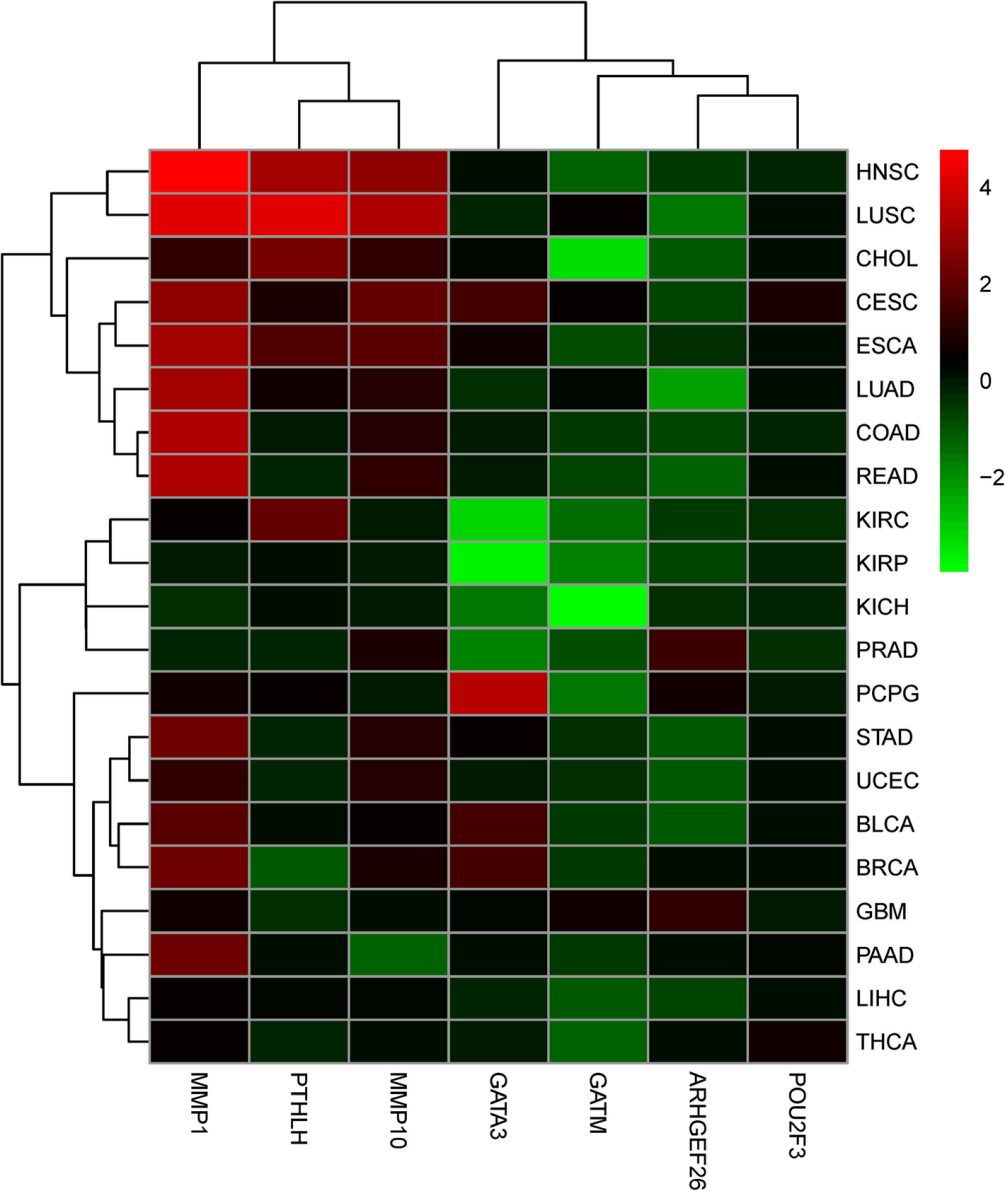
Differential expression for hub genes in 21 different TCGA cancer types

To understand the expression of hub genes in other cancers, we investigated the expression levels of 7 hub genes in primary patient tumors of 21 cancer types that have at least 3 paired adjacent normal samples (Fig. 6 and Supplementary Table 22). All hub genes except *POU2F3* showed significant differential expression in different cancer types. However, the direction of the altered expression varies for each gene and for each cancer type. Although *PTHLH*, *MMP1* and *MMP10* were mainly up-regulated in the 21 cancer types, the rest of the members *GATM* and *ARHGEF26*, were primarily down-regulated with a few exceptions. *GATA3* was both up-regulated and down-regulated in different cancer types.

### Association of hub gene with patient overall survival in pan-cancer

We further tested whether the expression of hub genes could also predict patient survival outcomes in other cancers. For the survival analysis, all 33 cancer types were tested with the Kaplan–Meier method. The results showed that each of the hub genes was significantly associated with the survival of several cancer types (Supplementary Table 23); however, the direction of the association varied depending on the member queried and the cancer type tested. More specifically, increased expression of *MMP1*, *MMP10* and *PTHLH* was mainly associated with increased survival disadvantage. *MMP1* predicted poor prognosis of patients with ACC, CESC, KICH, KIRP, LIHC, LUAD, MESO, PAAD, SARC and UVM. *MMP10* predicted poor prognosis for patients with LGG, LIHC, MESO and SARC. *PTHLH* predicted poor prognosis for patients with KIRP, LGG, LIHC, MESO, PAAD, THCA and UVM. In contrast, increased expression of *GATM*, *ARHGEF26* and *POU2F3* was primarily associated with survival advantage and predicted better survival. *GATM* predicted good prognosis for patients with ACC, KIRC, SARC and UCEC. *ARHGEF26* predicted good prognosis for patients with KIRC, OV and PAAD. *POU2F3* predicted good prognosis for patients with STAD and THCA. The rest of *GATA3* were associated with either survival advantage or disadvantage depending on the cancer types. In more detail, increased expression of *GATA3* predicted poor prognosis for patients with ACC, GBM, KIRP, LGG and UVM, but predicted survival advantage for patients with BLCA, SKCM and THYM. It is worth noting that only *GATA3* was significantly associated with the survival of SKCM, another skin cancer.

## Discussion

cSCC is a common malignant tumor that can be fatal if treatment is delayed. Clinical symptoms are not obvious in the early stages of cSCC, but cSCC is hard to cure in the late stages. Although new medical approaches have greatly improved the quality of life for patients with cancer, the 5-year survival rate of metastatic cSCC has not significantly improved. Studying the occurrence of cSCC at the genomic level, therefore, can help develop effective measures to prevent and inhibit cSCC metastasis. Here, we applied WGCNA to identify key modules and hub genes associated with cSCC and AK. We identified two modules associated with cSCC (module 5 from the GSE45216–98774 dataset and module 5 from the GSE108008 dataset). We considered hub genes present in both the GSE45216–98774 and GSE108008 datasets as validated hub genes. We thus identified and validated seven hub genes: *GATM*, *ARHGEF26*, *PTHLH*, *MMP1*, *POU2F3*, *MMP10* and *GATA3*. Three of them (*MMP1*, *MMP10*, and *PTHLH*) were also DEGs between cSCC and AK samples and between AK and normal samples, suggesting that they play continuous roles in AK and cSCC development. We identified two modules associated with AK in the GSE45216–98774 and GSE108008 datasets. However, no hub gene was validated for AK. These results provide new insights that will help explain the pathogenesis of cSCC, and the hub genes may become biomarkers or therapeutic targets for future accurate diagnosis and treatment of cSCC.

When we investigated the expression of hub genes in pan-cancer, we found great heterogeneity of the levels of hub gene expression among different tumor types. Although *MMP1*, *MMP10* and *PTHLH* were mainly up-regulated in the 21 cancer types, the other two members *GATM* and *ARHGEF26*, were primarily down-regulated. We further tested whether the expression of hub genes could also predict patient survival outcomes in pan-cancer, and found that the direction of association is also dependent on cancer type. In general, though, *MMP1*, *MMP10* and *PTHLH* were mainly associated with poor prognosis, indicating a tumor promoting role in most cancers. *GATM*, *ARHGEF26* and *POU2F3* were associated with better survival, and were recognized as tumor suppressors. The rest of *GATA3* had an antagonistic association with survival depending on the cancer types.

Many studies have reported that the seven hub genes are cancer-related and play roles in tumorigenesis and malignant phenotypes. *GATM* encodes a mitochondrial enzyme that catalyzes the biosynthesis of guanidinoacetate, the immediate precursor of creatine. This gene is mainly associated with renal cell cancer (RCC). A study found that BC039389-GATM chimeric read-through transcripts were up-regulated in RCC [20]. Assays performed in RCC-derived cell lines also revealed that two microRNAs targeted GATM for arginine metabolism [21]. *ARHGEF26* is a RhoG-specific guanine nucleotide exchange factor that plays a role in promotion of micropinocytosis. Glioblastoma tumors overexpress *ARHGEF26*, which favors glioma invasion [22]. A novel signaling pathway involving *ARHGEF26* regulates invadopodia disassembly in breast cancer cells [23]. The protein encoded by *PTHLH* is a parathyroid hormone, which regulates epithelial-mesenchymal interactions. *PTHLH* is up-regulated in oral squamous cell carcinoma (OSCC), head and neck squamous cell carcinoma (HNSCC), colon cancer, and hepatocellular carcinoma (HCC). *PTHLT* influences cell proliferation and cell cycle and is highly associated with metastasis [24–26].

Matrix metalloproteases (MMPs) are intriguing genes implicated in cancer progression, angiogenesis promotion, metastasis, and avoidance of immune surveillance. Many studies have noted that these genes are frequently up-regulated in cancers [27]. It has been shown that *MMP1* is associated with initiating malignant tumor formation leading to aberrant regulation of cell proliferation [28]. Some studies have implicated *MMP10* in colon and lung cancers. The *MMP10* level can serve as a marker of poor prognosis in patients with colon cancer [29]. It is required for lung cancer stem cell maintenance, tumor initiation, and metastatic potential [30]. *POU2F3* is primarily expressed in the epidermis and plays a key role in keratinocyte proliferation and differentiation. Its encoded protein is also a candidate tumor suppressor protein, and aberrant promoter methylation of this gene may play a role in cervical cancer. *POU2F3* has been reported to be used for recognizing different subtypes of small cell lung cancer (SCLC) [31, 32]. *GATA3* contains two GATA-type zinc fingers and is an important regulator of T-cell development, which plays an important role in endothelial cell biology. *GATA3* is a useful marker not only for mammary and urothelial but also for renal and germ cell tumors and mesotheliomas [33]. A recent genomic analysis of human breast cancer has revealed a high-frequency of mutation in *GATA3* in luminal tumors [34]. In an immunohistochemical expression assay of GATA3 protein in a wide variety of epidermal and cutaneous adnexal tumors, GATA3 exhibited positive staining in most cases [35].

*MMP1* and *GATA3* have shown their demonstrated links with cSCC. Invasive cSCC have significantly higher *MMP1* mRNA and protein levels than non-invasive cSCC [36]. A report has shown that decreased GATA3 protein immunohistochemical staining is associated with cSCC progression [37]. As for *GATM*, *ARHGEF26*, *PTHLH*, *POU2F3*, and *MMP10*, which are relatively new molecules, there are few reports on their role in cSCC. Nevertheless, they play an important role in cSCC tumorigenesis, with significant differences between cSCC and AK. Understanding their roles in cSCC requires further research.

Using two datasets containing normal, AK, and cSCC samples, we identified and validated seven hub genes related to cSCC. We acknowledge that this study has some limitations and shortcomings. First, the hub genes for AK development were not verified. Second, the clinical parameters and prognosis were not well analyzed for cSCC samples due to the availability of data.

## Conclusion

We applied WGCNA to construct co-expression networks and explore the gene expression in cSCC. We found seven hub genes (*GATM*, *ARHGEF26*, *PTHLH*, *MMP1*, *POU2F3*, *MMP10* and *GATA3*) that played important roles in cSCC tumorigenesis. Among them, three genes (*MMP1*, *MMP10*, and *PTHLH*) may play continuous roles in AK and cSCC development. Abnormalities in the pathway of focal adhesion may be one of the causes of cSCC pathogenesis. These seven hub genes may provide a better understanding of tumorigenesis mechanisms in patients with cSCC. Moreover, these hub genes may serve as prognostic biomarkers and therapeutic targets in the future.

## Supplementary Information

**Additional file 1: Supplementary Table 1.** Genes in the module 5 for cSCC in the GSE45216–98774 dataset. **Supplementary Table 2.** Genes in the module 23 for AK in the GSE45216–98774 dataset. **Supplementary Table 3.** Genes in the module 9 for normal sample in the GSE45216–98774 dataset. **Supplementary Table 4.** Genes in the module 5 for cSCC in the GSE108008 dataset. **Supplementary Table 5.** Genes in the module 10 for AK in the GSE108008 dataset. **Supplementary Table 6.** Genes in the module 7 for normal sample in the GSE108008 dataset. **Supplementary Table 7.** Overrepresented GO terms in the module 5 relevant for cSCC in the GSE45216–98774 dataset. **Supplementary Table 8.** KEGG functional enrichment of genes in the module 5 relevant for cSCC in the GSE45216–98774 dataset. **Supplementary Table 9.** KEGG functional enrichment of genes in the module 23 relevant for AK in the GSE45216–98774 dataset. **Supplementary Table 10.** Overrepresented GO terms in the module 9 relevant for normal tissue in the GSE45216–98774 dataset. **Supplementary Table 11.** KEGG functional enrichment of genes in the module 9 relevant for normal tissue in the GSE45216–98774 dataset. **Supplementary Table 12.** Overrepresented GO terms in the module 5 relevant for cSCC in the GSE108008 dataset. **Supplementary Table 13.** KEGG functional enrichment of genes in the module 5 relevant for cSCC in the GSE108008 dataset. **Supplementary Table 14.** Overrepresented GO terms in the module 10 relevant for AK in the GSE108008 dataset. **Supplementary Table 15.** KEGG functional enrichment of genes in the module 10 relevant for AK in the GSE108008 dataset. **Supplementary Table 16.** Overrepresented GO terms in the module 7 relevant for normal tissue in the GSE108008 dataset. **Supplementary Table 17.** KEGG functional enrichment of genes in the module 7 relevant for normal tissue in the GSE108008 dataset. **Supplementary Table 18.** Differentially expressed genes when cSCC compared with AK samples in dataset GSE45216–98774. **Supplementary Table 19.** Differentially expressed genes when AK compared with normal samples in dataset GSE45216–98774. **Supplementary Table 20.** Differentially expressed genes when cSCC compared with AK samples in dataset GSE108008. **Supplementary Table 21.** Differentially expressed genes when AK compared with normal samples in dataset GSE108008. **Supplementary Table 22.** Differential expression in 21 different TCGA cancer types. **Supplementary Table 23.** Association of hub gene expression with patient overall survival for different cancer types.

## Data Availability

All data generated or analyzed during this study are included in this published article.

## Abbreviations

DEGs: Differentially expressed genes
cSCC: Cutaneous squamous cell carcinoma
AK: Actinic keratosis
GO: Gene Ontology
KEGG: Kyoto Encyclopedia of Genes and Genomes
WGCNA: Weighted gene co-expression network analysis
FDR: False discovery rate
ACC: Adrenocortical carcinoma
BLCA: Bladder Urothelial Carcinoma
BRCA: Breast invasive carcinoma
CESC: Cervical squamous cell carcinoma and endocervical adenocarcinoma
CHOL: Cholangio carcinoma
COAD: Colon adenocarcinoma
DLBC: Lymphoid Neoplasm Diffuse Large B-cell Lymphoma
ESCA: Esophageal carcinoma
GBM: Glioblastoma multiforme
HNSC: Head and Neck squamous cell carcinoma
KICH: Kidney Chromophobe
KIRC: Kidney renal clear cell carcinoma
KIRP: Kidney renal papillary cell carcinoma
LAML: Acute Myeloid Leukemia
LGG: Brain Lower Grade Glioma
LIHC: Liver hepatocellular carcinoma
LUAD: Lung adenocarcinoma
LUSC: Lung squamous cell carcinoma
MESO: Mesothelioma
OV: Ovarian serous cystadenocarcinoma
PAAD: Pancreatic adenocarcinoma
PCPG: Pheochromocytoma and Paraganglioma
PRAD: Prostate adenocarcinoma
READ: Rectum adenocarcinoma
SARC: Sarcoma
SKCM: Skin Cutaneous Melanoma
STAD: Stomach adenocarcinoma
TGCT: Testicular Germ Cell Tumors
THCA: Thyroid carcinoma
THYM: Thymoma
UCEC: Uterine Corpus Endometrial Carcinoma
UCS: Uterine Carcinosarcoma
UVM: Uveal Melanoma
